# Modulating Laying Hens Productivity and Immune Performance in Response to Oxidative Stress Induced by *E. coli* Challenge Using Dietary Propolis Supplementation

**DOI:** 10.3390/antiox9090893

**Published:** 2020-09-21

**Authors:** Ahmed O. Abbas, Abdulaziz A. Alaqil, Hossam S. El-Beltagi, Hanaa K. Abd El-Atty, Nancy N. Kamel

**Affiliations:** 1 Department of Animal and Fish Production, College of Agricultural and Food Sciences, King Faisal University, P.O. Box 420, Al-Ahsa 31982, Saudi Arabia; aalaqil@kfu.edu.sa; 2 Department of Animal Production, Faculty of Agriculture, Cairo University, Gamma St., Giza, Cairo P.O. Box 12613, Egypt; 3 Department of Agricultural Biotechnology, College of Agricultural and Food Sciences, King Faisal University, P.O. Box 420, Al-Ahsa 31982, Saudi Arabia; helbeltagi@kfu.edu.sa; 4 Department of Biochemistry, Faculty of Agriculture Cairo University, Gamma St., Giza, Cairo P.O. Box 12613, Egypt; 5 Department of Poultry breeding, Animal Production Research Institute, Agricultural Research Center, Dokki, Giza P.O. Box 12611, Egypt; hanaa.amin@arc.sci.eg; 6 Department of Animal Production, National Research Center, El Buhouth St., Dokki, Giza, Cairo P.O. Box 12622, Egypt

**Keywords:** propolis, *E. coli*, inflammation markers, lymphocyte proliferation, leucocyte cell viability, Foxo3, laying hen

## Abstract

Propolis (PR) is a resin product of bee colonies that has rich bioactive antioxidant and bactericidal compounds. Endotoxin, a byproduct of bacterial growth, is reported to cause progressive induction of endogenous oxidative stress and has negative impacts on individual health and wellbeing. Hereby, we investigated the ability of PR to alleviate the oxidative stress and immunosuppression imposed by avian pathogenic *Escherichia coli* using laying hen as a based model. In this study, PR was dietary supplemented to hens for 4 weeks at a concentration of 0.1%. At the beginning of the 4th week of the experiment, hens from control and PR treatment were injected with *E. coli* (O157:H7; 10^7^ colonies/hen) or saline. The results showed significant (*p* < 0.05) negative impact of *E. coli* challenge on antioxidant status, immune response and productive performance. PR supplementation reduced (*p* < 0.05) inflammation markers levels (tumor necrosis factor α (TNFα) and interleukin 1β (IL-1β)) and plasma corticosterone concentration. The antioxidant status was ameliorated with dietary PR supplementation to challenged hens, showing significant (*p* < 0.05) reduction in malondialdehyde (MDA) levels and increasing total antioxidant capacity (TAC) concentrations. Cell mediated, as well as, humeral immune response improved significantly (*p* < 0.05) with dietary PR verified by the enhancement of T- and B-lymphocyte proliferation and the positive respond to phytohemagglutinin (PHA). Leucocyte cells viability increased significantly and the apoptotic factor forkhead box O3 (Foxo3) was reduced with PR supplementation. The current study revealed that dietary PR supplementation can effectively be used as an organic feed additive to overcome the endogenous oxidative stress induced by endotoxins challenge.

## 1. Introduction

Recently, oxidative stress has become a major concern as a life threatening and a chronic- disease mediator [1,2,3]. The reactive oxygen or nitrogen species (ROS, RNS) are normally generated during the respiratory chain reaction in the mitochondria. Nevertheless, the excessive production of such ROS and RNS induces oxidative stress, homeostasis imbalance and subsequent pathological conditions [4]. Bacterial infection activates immune cells and induces inflammation, which is considered as being responsible for endogenous free radical production [5]. T helper cell (Th1) mediates the immune system protection to specific pathogens by the secretion of different pro-inflammatory cytokines such as interferon gamma (IFN-γ), interleukin-2 (IL-2), tumor necrosis factor α (TNF-α), lymphotoxin and granulocyte-macrophage colony-stimulating factor [6]. The excessive pro-inflammatory cytokines release and the excess formation of ROS/RNS induce uncontrolled tissue damage. Thus, in order to alleviate the negative impact of bacterial infection and the oxidative-load imposed under such conditions, antioxidant and antimicrobial agents are required. However, multidrug-resistance pathogens have become an increasing national and international concern [7,8,9]. Food-borne diseases and antibiotic-resistance bacteria impose increasing hazards on human health [10,11]. Pollution due to the presence of antibiotic residues contributed to the increasing occurrence of multidrug-resistance microbes and increased abundance of antibiotic-resistance genes [12,13].

*Escherichia coli* (*E. coli*) is a Gram negative, facultative anaerobic bacterium that naturally lives not only in chicken and human guts but also in other warm-blooded animals. *E. coli* is considered to be the primary bacterial species that leads to the spreading of antibiotic-resistance gene in poultry farms [14]. Infection with avian pathogenic *E. coli* strains (APEC) negatively affected both humeral and cellular immune responses of broiler chickens [15]. Moreover, oral administration of *E. coli* caused an increase in gut-pathogenic bacterial counts and a reduction in beneficial bacterial counts [16]. Furthermore, in Egypt, Ramadan et al. [17] demonstrated the existence of shared antimicrobial resistances between *E. coli* isolated from retail chicken carcasses and humans. Recently, there has been an increasing interest in replacing antibiotics with an alternative variety of natural products, such as medicinal plants [15,18] and probiotics [18,19,20,21], to ameliorate poultry antioxidant status, immune response, growth performance and egg production.

Propolis (PR) is a resinous material produced by honeybees (*Apis mellifera*) from beeswax and resins collected from plants. Honeybees use PR as a social immune defense against different kinds of parasites and pathogens [22]. The presence of antimicrobial, antioxidant and cytotoxic phytochemicals in PR ethanol extract were reported [23]. Polyphenols and flavonoids are major PR compounds that were found to be related to their antioxidant effect in a dose-dependent manner [24,25,26]. PR extraction and its derivative reported to have antioxidant [27], antimicrobial [28], anti-inflammatory [29,30] and cytotoxic [30] activities. In poultry, PR has been recently used for its antioxidant, antimicrobial and growth promoting ability [31,32,33,34,35]. However, the interactions among in vivo antioxidant, antimicrobial, anti-inflammation and immune-modulation effects of PR specially regulating cell death program of immune cells and T and B lymphocyte proliferation under endotoxin stress have not been fully investigated. Thus, the present study was designed to evaluate the phyto-therapeutic ability of PR to overcome the negative effects of endotoxins after *E. coli* challenge and its subsequent induction of oxidative load on laying hen. Oxidative status, immune response, gut morphology and productive performance were the main studied aspects.

## 2. Materials and Methods

### 2.1. Birds and Ethical Statement

After *E. coli* challenge, scheduled observations were set for evaluating the pathogenic stress symptoms progression on the wellbeing of the experimental birds. Challenged hens were closely monitored to detect any signs of distress (i.e., decreased appetite, listless, ruffled feathers, beak fluid discharge, breathing difficulty or fever). Consequently, to minimize bird suffering, if at least one of the above-mentioned signs appeared, the body temperature was immediately taken. If the temperature elevated to be 43.5 °C or higher, cervical dislocation was used to allow a humane endpoints. Ethical approval, for all the practiced experimental protocols, was obtained from Medical Research Ethics Committee, National Research Center (NRC-MREC; number, 20/129) according to Egyptian Network of Research Ethics Committee (ENREC) regulations.

### 2.2. Experimental Design and Birds Management

A total of 240 healthy H&N Brown Nick layer chickens at 40-weeks of age were recruited and randomly assigned into two equal dietary treatments. The dietary treatments lasted for four weeks, where the control groups received a basal diet, while the propolis-supplementation groups received the basal diet supplemented with 0.1% propolis (1 g/kg diet). At the beginning of the 4th week of the experiment, hens from the control and propolis supplementation groups were further divided into two sub-groups each (5 replicates, 12 hens each). One of each sub-group was challenged with single intraperitoneal (ip) injection of *E. coli* O157:H7 (10^7^ colonies/hen dissolved in 0.5 mL of sterile saline), while the remaining two sub-groups were ip injected with 0.5 mL sterile saline. Consequently, the experimental groups were assigned as follow; (1) control with saline injection (C); (2) control with *E. coli* challenge (EC); (3) propolis supplementation with *E. coli* challenge, (PR + EC) and (4) propolis supplementation with saline injection (PR).

The basal diet was prepared to meet the nutrition requirements of hens under the new management guide of H&N^®^ International (https://www.hn-int.com/). Diet and fresh water were offered ad libitum. Hens were housed in laying cages with 3 hens per cage in an open poultry house. The light regimen was set to be 16L:8D throughout the experimental period. The *E. coli* used in this study was brought from the U.S. Department of Health and Human Services (Washington D.C., USA) through Cairo Microbiological Resources Center (Cairo, Egypt). PR was purchased, in a powder form, from the apiary of the Faculty of Agriculture Cairo University, located in Giza governorate, Egypt. PR was collected during March 2020, and was stored in dark sealed glass bottle until its use.

### 2.3. Blood Sample Collection and Preparation

At the end of 44 weeks of age, 10 blood samples per treatment (2 samples per replicate) were collected from the brachial vein (5 mL/hen) using heparinized syringes. One mL of each blood sample was centrifuged at 2000× *g* for 10 min at 4 °C to separate and store plasma at −20 °C to determine corticosterone concentration. Meanwhile, the 4 mL of each sample was used to isolate the peripheral blood mononuclear cells (PBMCs) as reported by Mehaisen et al. [36]. Briefly, the PBMCs were washed twice using cell culture medium (Gibco RPMI 1640 Medium, Thermo Fisher Scientific, Waltham, MA, USA), to remove the residual platelets, and re-suspended with phosphate-buffered saline (PBS) (pH 7.2). Then, 1 mL of cell suspension was centrifuged for 20 min at 1030× *g* for 20 min and the aggregated granules obtained were stored at −70 °C for further analyses (TNF-α, interleukin 1β (IL-1β), malondialdehyde (MDA), total antioxidant capacity (TAC), super oxide dismutase (SOD) and forkhead box O3 (Foxo3) expression).

### 2.4. Productive Performance

Daily egg numbers, egg weights and feed intake (g/hen/d) were recorded for each experimental group for one week post *E. coli* challenge. Feed conversion was then calculated, as g feed intake/g egg mass.

### 2.5. Propolis Phenolic Content and Free Radical Scavenging Activity

Propolis total phenolic acids and flavonoids contents were analyzed using high-performance liquid chromatography (HPLC) (Agilent 1260 Infinity, Santa Clara, CA, USA) as previously described [31]. All the chemicals used were HPLC grade. Sample separation was carried out using 20 μL sample injection volume with flow rate of 1 mL/min. The retention time of sample against reference standard was used to identify and quantify the eluted components. The absorption wavelength was set at 284 nm.

**Figure 1:**
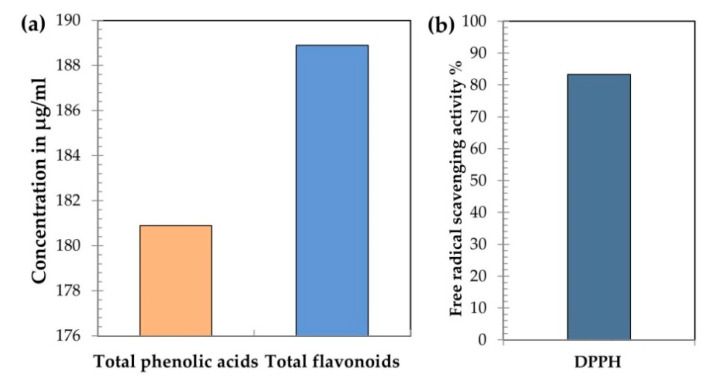
Polyphenols content measured by HPLC (**a**) and free radical scavenging activity as DPPH% (**b**) of propolis supplemented to hen diet.

The antioxidant activity of propolis was measured using the 2,2-diphenyl-1-picrylhydrazyl (DPPH) assay according to Shimada et al. [37]. Briefly, 1 mL of the solution (0.1 mM of DPPH dissolved in methanol) was added to 3 mL of PR solution at different concentrations (0.78–100 µM). The mixture was well-stirred and kept in darkness at room temperature. After 30 min of incubation, samples absorbance was detected at 517 nm according to Oktay et al. [38]. Propolis phenolic and flavonoid contents as well as free radical scavenging activity (DPPH) are presented in Figure 1a,b.

### 2.6. Physiological Parameters

#### 2.6.1. Inflammation Markers and Antioxidant Parameters in PBMCs

The stored PBMCs were washed and re-suspended with 1 mL of PBS, kept on ice for one minute and then sonicated for another one minute. The obtained homogenate was centrifuged at 1030× *g* and 4 °C for 15 min. The collected supernatants were used to quantify the inflammatory cytokines and antioxidant parameters levels. TNF-α and IL-1β levels were measured using chicken specific enzymatic-linked immunosorbent assay (ELISA) diagnostic kit supplied by MyBioSource, San Diego, CA, USA (cat# MBS2509660 and MBS761055, respectively). While, malondialdehyde, total antioxidant capacity and superoxide dismutase levels were determined using quantitative colorimetric assay kits supplied by Abcam, Cambridge, UK (cat# ab118970, ab65329 and ab65354 respectively).

#### 2.6.2. Plasma Corticosterone Hormone Assay

Plasma corticosterone levels were quantified (*n* = 10, 2 samples per replicate) using ELISA kits specific for chicken (cat# MBS701668; MyBioSource, San Diego, CA, USA). According to the manufacturer, the intra-assay and inter-assay coefficient of variability were <8% and <10%, respectively, with dynamic range of 0.5 to 20 ng/mL.

### 2.7. Immunological Parameters

#### 2.7.1. Wattle Thickness in Response to Phytohemagglutinin-P (PHA) Antigen Injection

Wattle thickness response to phytohemagglutinin-P (PHA) antigen injection was performed as described by Edelman et al. [39]. Briefly, a solution of PHA was prepared by dissolving 100 µg in 0.1 mL of sterile PBS buffer. The hens were injected (*n* = 10; 2 birds per replicate) in the center of the wattle with 50 µL of the PHA solution. To assess cell-mediated immune response, wattle thickness was measured using a caliper (Schnelltester automatic caliper) both before and 24 h after PHA injection.

#### 2.7.2. Total WBC Count and Heterophile/Lymphocyte (H/L) Ratio Determination

Total leukocyte counts in the whole blood for 10 samples per treatment (2 samples per replicate) were performed manually using a hemocytometer slide as described by Gehad et al. [40]. Meanwhile, H/L ratio was determined manually according to the avian guidelines [41], using Hema-3 stain (cat# 22–122,911, Fisher Scientific, Pittsburg, PA, USA). After staining, H/L ratios were calculated by the differential leukocyte counting (200 leukocytes; heterophil against lymphocyte) which was performed by using light microscope at a magnification of 100× with oil immersion.

#### 2.7.3. Peripheral T- and B-Lymphocyte Proliferation

The stimulating index (SI) of T- and B-lymphocyte proliferation was calculated according to Mehaisen et al. [34]. The method, in brief, started with washing PBMCs (*n* = 10; 2 samples per replicate) in RPMI 1640 culture medium and viable lymphocytes were detected (using Trypan Blue dye; cat# T8154, Sigma-Aldrich, St. Luis, MO, USA). The viable cells were then plated in triplicate at 6 × 10^6^ cells per well using 96-well plate. Then, 50 μL of either concanavalin-A at a concentration of 45 μg/ mL (cat# C5275, Sigma-Aldrich, St. Luis, MO, USA) or lipopolysaccharide at a concentration of 10 μg/mL (cat# L4391, Sigma-Aldrich, St. Luis, MO, USA) was added to stimulate T-cells and B-cells proliferation, respectively. Meanwhile, 50 μL of RPMI-1640 medium (Gibco, Thermo Fisher Scientific, Waltham, MA, USA) was added to the un-stimulated cells (control). Cells were incubated with 5% CO_2_ at 42 °C for two days. Afterwards, 15 μL of 3-(4,5-Dimethyl-2-thiazolyl)-2,5-diphenyl-2H-tetrazolium bromide (MTT) (cat# M2128, Sigma-Aldrich, St. Luis, MO, USA) was added at a concentration of 5 mg/mL to each well, then cells were incubated at 42 °C. Four hours later after incubation, 100 μL of 10% sodium dodecyl sulfate, dissolved in 0.04 M HCl, was added per well. The absorbance was detected at 570 nm using ChroMate Microplate Reader-4300 (Awareness Technology Inc., Palm City, FL, USA). T- and B-lymphocytes SI was calculated as the optical density ratio of stimulated cells to un-stimulated control cells.

### 2.8. Small Intestine Histomorphology

Ileum sections of the small intestinal samples were collected from hens after cervical dislocation (*n* = 5; one sample per replicate) as described by Mehaisen et al. [33]. Samples were thoroughly washed and soaked for 72 h in 10% neutral buffered formalin. Cross sections (thickness was 3–5 μm) were obtained, by a rotatory microtome, and stained using general staining method with Harris hematoxylin and eosin stain [42]. The villus height and crypts depth of sample sections were examined and determined under light microscope at 40× magnification using image analysis software (Leica Microsystems, Wetzlar, Germany).

### 2.9. Leucocyte Cell Viability

Leucocyte cell viability (*n* = 10; 2 samples per replicate) was measured using a modified MTT assay [43]. Briefly, 5 mg of tetrazolium salt MTT (Serva, Heidelberg, Germany) was dissolved in 1 mL of AIM-V medium. Using a 98-well plate, 100 μL of cell culture medium was supplemented with 25 μL of (5 mg/mL) MTT solution, and the cells were then incubated for 4 h at 37 °C. After incubation, the plates were centrifuged for 10 min at 600× *g*, and the incubation medium was aspirated. Acidified isopropyl alcohol solution (0.04 N HCl) was added to each well (100 μL) and then the absorbance of formazan was measured at 570 nm using ELISA reader.

### 2.10. Foxo3 Expression in PBMCs

The expression of forkhead box O3 in PBMCs supernatant was analyzed using western blotting method. The primary antibodies of β-actin (1:1000 dilution, Santa Cruz Biotechnology, Dallas, TX, USA) and Foxo3 (1:500 dilution, cat# AB12162, Abcam, San Francisco, CA, USA) were employed while the Flag-Foxo3 chicken protein source was bought from LMAI-BIO (Shanghai, China). The polyvinylidene fluoride (PVDF) membranes were incubated overnight at 4 °C with the primary antibodies. The incubation followed by washing twice with Tris-buffer saline with 0.1% (*v*/*v*) Tween 20. Afterwards, the PVDF membrane was incubated for 2 h with horseradish peroxidase-conjugated immunoglobulin G (IgG) antibody (1:2000 dilution, Santa Cruz Biotechnology, Dallas, TX, USA) at room temperature. Chemiluminescence detection method was then conducted to obtain results.

### 2.11. Statistical Analysis

The obtained data were analyzed by two-way ANOVA using the general linear model (GLM) of SAS Software Package [44]. The main effect of dietary propolis supplementation (PR), *E. coli* challenge (EC) and their interaction (PR × EC) on the different quantitative parameters measured were assessed. When significant differences due to PR, EC or PR × EC were detected, Least Significant Difference (LSD) test was performed to determined significance among experimental groups. Statistical difference was set at *p* < 0.05 and results were expressed as LSM ± SEM.

## 3. Results

### 3.1. Productive Performance

Productive performance of laying hens was influenced by *E. coli* challenge and dietary PR supplementation (Table 1). The presented data showed a significant (*p* < 0.05) reduction in egg number and egg weight by 36% and 12.6%, respectively, in EC group compared to C group. Furthermore, feed intake was reduced (*p* < 0.05) by 25% and feed conversion was impaired (*p* < 0.05) by 35% with *E. coli* challenge compared to C group. However, dietary PR supplementation was able to (*p* < 0.05) reduce the negative impact of *E. coli* challenge on egg number, egg weight and feed intake by 20%, 7% and 18%, respectively, compared to the laying hens fed on basal diet and challenged with *E. coli*. However, there was no significant change in productive performance between laying hens in PR and C groups.

### 3.2. Inflammation Markers and Antioxidant Status

The changes in TNF-α, IL-1β, MDA, TAC and SOD levels in PBMCs as well as plasma corticosterone concentrations in response to *E. coli* or saline injection in laying hens fed on basal diet or basal diet supplemented with PR for four weeks are shown in Table 2. *E. coli* injection induced inflammation by significantly (*p* < 0.05) increasing TNF-α, IL-1β and plasma corticosterone level by 1.9, 2.9 and 2.7 folds, respectively, compared to C group. The negative effect of *E. coli* challenge was alleviated by providing the laying hens a diet supplemented with PR. A significant reduction (*p* < 0.05) was noticed in TNF-α by 13% and in both IL-1β and plasma corticosterone by 37%. Meanwhile, there were no significant differences of the studied inflammatory markers levels in PR group compared to C group.

Changes in oxidative status were identified in PBMCs by screening MDA, TAC and SOD levels (Table 2). The MDA levels increased (*p* < 0.05) by 2.2-folds and TAC decreased significantly (*p* < 0.05) by 34% while the SOD concentration did not change in EC group compared to C group. However, adding PR to the basal diet minimized (*p* < 0.05) the elevation of MDA concentration induced by *E. coli* challenge by 45% in PR + EC group and retuned back to the normal level compared to control. Furthermore, data showed that dietary PR supplementation in PR group caused a significant (*p* < 0.05) improvement in the antioxidant status by increasing the concentration of TAC and SOD, by 84% and 20%, respectively, compared to C group.

### 3.3. Immunological Performance

The data in Table 3 demonstrated that laying hens fed on a basal diet and exposed to *E. coli* challenge showed suppression of all immunological measured parameters compared to other experimental groups, while the dietary PR supplemented group was able to reverse the negative effects induced by *E. coli* challenge. Total white blood cells (TWBCs) were reduced (*p* < 0.05) by 35.5% and 15.8%, while heterophile/lymphocyte (H/L) ratio was increased (*p* < 0.05) by 2.6 and 1.8 folds in EC and PR + EC groups, respectively, compared to C group. Wattle thickness in response to PHA injection was decreased (*p* < 0.05) by 20% in EC group compared to C group. Furthermore, the lymphocyte proliferation of T-cells was suppressed (*p* < 0.05) by 2.7-folds and 29.5% and B-cell proliferation was inhibited (*p* < 0.05) by 2-folds and 15.8%, respectively, in EC and PR + EC groups, compared to control. On the other hand, dietary propolis supplementation in PR group significantly elevated these immunological parameters except for H/L ratio compared to C group.

### 3.4. Small Intestines Histomorphology

**Figure 2:**
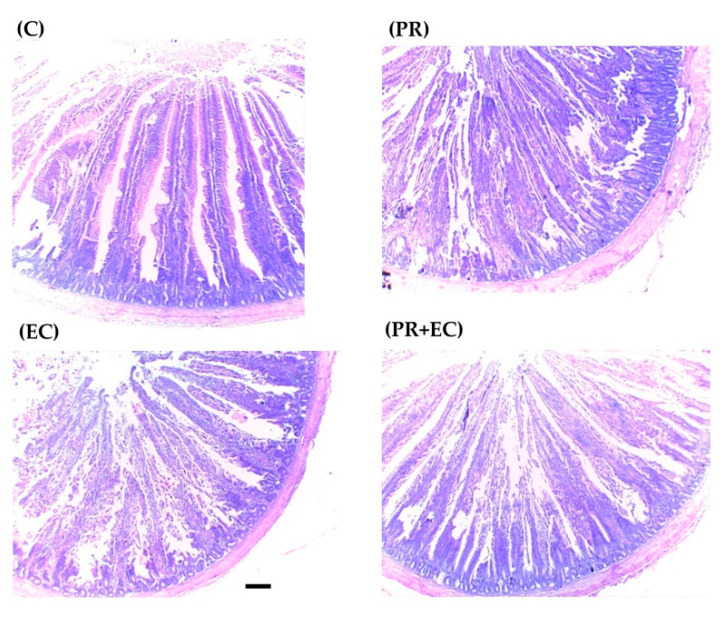
Histomorphological images of small intestines of laying hens (Scale bar 100 μm): (**C**) laying hens received a basal diet and injected with saline (control); (**EC**) laying hens received a basal diet and injected with *E. coli* (10^7^ colonies/hen); (**PR + EC**) laying hens received a basal diet supplemented with propolis at 1 g/kg feed and injected with *E. coli* (10^7^ colonies/hen) and (**PR**) laying hens received a basal diet supplemented with propolis at 1 g/kg feed and injected with saline.

The change of villi height, crypt depth and their ratio in response to *E. coli* challenge and dietary PR supplementation in laying hens are presented in Table 4. *E. coli* challenge showed no significant (*p* ≥ 0.05) changes in villi height or crypt depth compared to C group. However, dietary PR supplementation, with or without *E. coli* challenge, caused a significant increase (*p* < 0.05) of villi height (Figure 2). Meanwhile, there was no significant effect on crypt depth or their ratio compared to C group.

### 3.5. Leucocyte Cell Viability and Foxo3 Expression

**Figure 3:**
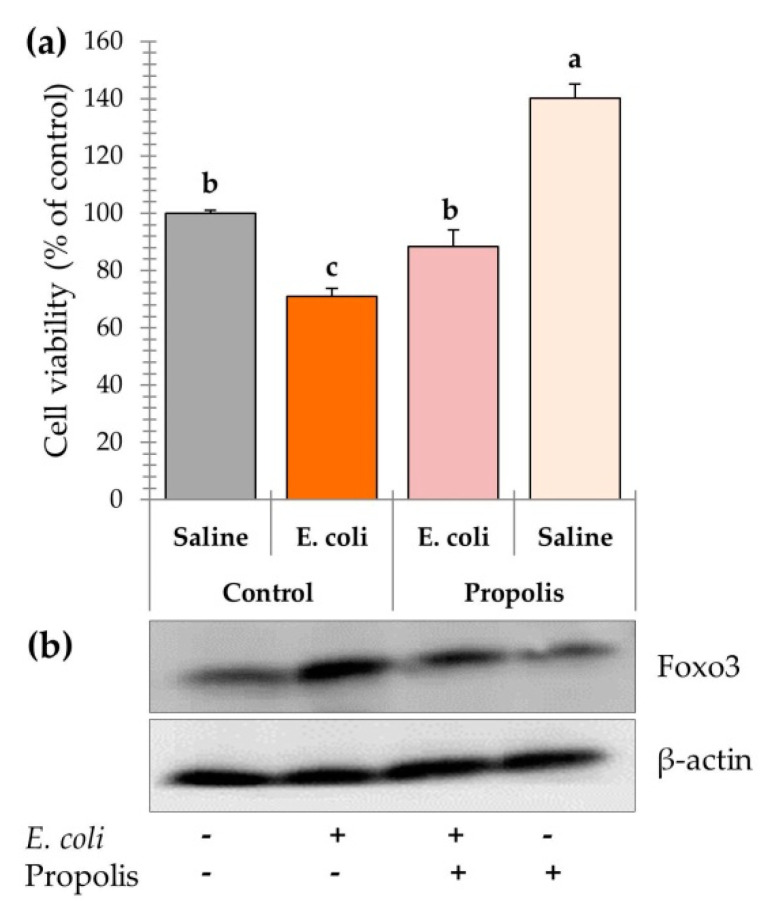
The effect of dietary propolis supplementation (1 g/kg diet) and *E. coli* challenge (10^7^ colonies/hen) on (**a**) leucocyte cell viability percentage and (**b**) the levels of Foxo3 expression in PBMCs. Column with different above letter significantly differ (*p* < 0.05).

Leucocyte cell viability percentage (CV%) was significantly decreased (*p* < 0.05) with *E. coli* challenge (Figure 3a) in EC group. Whereas, dietary PR supplementation was able to significantly (*p* < 0.05) alleviate the negative effect induced by *E. coli* challenge in PR + EC group on CV% and returned back to the normal level in C group. On the other hand, dietary PR supplementation in PR group significantly (*p* < 0.05) increased CV% by 40% compared to C group. Moreover, the level of Foxo3 protein expression was influenced by *E. coli* challenge and dietary PR supplementation (Figure 3b). *E. coli* challenge induced over-expression of Foxo3 in EC group compared to C group. However, dietary propolis supplementation in PR and PR + EC groups normalized the Foxo3 expression to similar levels of the C group. It is worth to notice that the concentration of Foxo3 protein negatively correlated with CV% values.

## 4. Discussion

Pathogenic bacterial infection imposes a huge physiological stress on birds. The negative impact of APEC infection on poultry production is mediated by endotoxin release [45] and oxidative stress generation [46]. The present results showed impairment in laying hen production with *E. coli* administration. Reduction of egg number and egg weight, in addition to low feed intake and impaired feed conversion were the major negative effects of *E. coli* administration (Table 1). Inoculation of APEC, whether intraperitoneal or intra-uterine, reported to cause a significant reduction in egg production efficiency in White Leghorn hens [47]. This negative effect could be in part mediated by the fact that *E. coli* induced excess production of ROS/RNS, exceeding the ability of the antioxidant defense system to cope with, which directly increase cell damage and subsequently reduce production [48]. da Rosa et al. [49] reported that the negative effect of *E. coli* infection on chicken growth performance was mediated by oxidative stress indicated with remarkable elevation in serum and hepatic ROS formation. On the other hand, propolis supplementation was reported to improve egg production quantitatively and qualitatively in laying Japanese quail subjected to heat induced oxidative stress [34]. The improvement in egg production with propolis supplementation may be justified by its antioxidant [50] and antimicrobial activities [51]. PR supplementation reported to increase egg production rate and shell weight as well as improve egg quality and feed conversion ratio in laying hen [52]. Moreover, Seven [53] reported that dietary PR supplementation to laying hen subjected to oxidative stress, induced by cyclic heat stress, was able to increase growth performance, nutrient digestibility, egg weight and egg shell weight as well as decrease mortality rate. The possible mechanisms of PR influencing layer productively under APEC challenge is partially mediated by its antimicrobial ability to reduce pathogenic bacterial growth in the chicken lower gastrointestinal tract [54] and improve Ca absorption and digestibility [55] due to its acid derivatives contents. Further, positive impacts of PR on layer performance may be attributed to the palatable components existed in PR such as resin, vanillin, wax and honey [56], and these components could give beneficial results when hens are kept under stress conditions [53]. 

Pro-inflammatory cytokines are peptides released from immune cells, mainly macrophage and T-lymphocytes, upon activation [57]. When birds confront a pathogen, inflammation is a mechanism of the innate immunity to contain the bacterium infection and reduce pathogenic threatens [58]. The significant increase in TNF-α, IL-1β, H/L ratio and plasma corticosterone levels with *E. coli* challenge indicate the induction of inflammation and stress responses (Table 2 and Table 3). Mehaisen et al. [59] reported that intra-veins *E. coli* injection increased TNF-α and IL-1β genes expression by two and three folds, respectively, in the brain and liver tissues of the laying hens. It is fundamental to note that the elevation in cytokines production induces the hypothalamic-pituitary-adrenal axis which consequently increases plasma corticosterone concentration as previously described [60,61,62]. Mol et al. [63] suggested that the immune response of chicken lung epithelial cell line to APEC invasion depends on the up-regulation of IL-8 expression which subsequently attracts macrophages and heterophils to the infection cite. In addition, chicken macrophage-like cells showed up-regulation of different cytokines gene expression (IL-8, IL-6, IL-1β and IL-10) when incubated with APEC [62]. Blocking pro-inflammatory cytokines was suggested to potentially mitigate the negative effect of *E. coli* inducing intestinal inflammation in rat [64]. Propolis supplementation was reported to have the capability of reducing inflammation, suppressing cytokines production of immune cells as well as reducing corticosterone level [33,65]. The anti-inflammatory properties of propolis are mediated by its ability to reduce oxidative stress, [66] in addition to have hyaluronidase inhibitory activity [26].

Stress imposed by infectious disease induces imbalance oxidative status with excess production of ROS/RNS and lipid peroxidation. Significant increase in MDA with low TAC concentrations was observed when laying hens were challenged with *E. coli* (Table 2). Ayala et al. [67] linked between high levels of free radical and ROS formation and lipid peroxidation. In rats, intraperitoneal injection of LPS driven from *E. coli* induced lipid peroxidation and reduced glutathione, glutathione-S-transferase and SOD activities as well as TAC with a progressive increasing effect over time [68]. The cell antioxidant defense includes antioxidant enzymes (e.g., SOD, GPx and catalase) and free radical scavenging antioxidants (e.g., vitamin E and C) in addition to other enzymes and proteins involved in the repair or removal of the damaged molecules (e.g., heat shock proteins, DNA-repairing enzymes and proteasomes) [48]. Yangi et al. [69] suggested that the protective effect of propolis to LPS-induced endotoxin stress was mediated by attenuating the inflammatory response and its subsequent ROS production. Previous researches reported that propolis has a high content of phenolic acids and flavonoids [28] in addition to high antioxidant activity [31]. Furthermore, the antioxidant activities of different propolis extracts depend on their phenolic content [24,70]. This data was confirmed by the presented HPLC and DPPH results of the dietary supplemented propolis (Figure 1a,b). In the present study, the high phenolic content and free radical scavenging activity found in propolis suggested a high affinity for oxidative reduction which causes the decrease observed in MDA and the increase in TAC. It can be inferred from the present result that propolis supplementation improved oxidative status by mediating the non-enzymatic antioxidant compounds rather than increasing the concentration of endogenous antioxidant enzymes (i.e., SOD).

Villi height and crypt depth of small intestine were not affected by *E. coli* administration compared to the C group (Table 4). Although, direct negative effect of oral administration of *E. coli* on gut morphology was reported with decreasing both villus width and height [16], other research reported no effect of *E. coli* challenge on villi height or crypt depth [71]. Nevertheless, PR supplementation increased villi height and crypt depth significantly (Table 4 and Figure 2). Dietary propolis supplementation, at 1 g/kg feed mixture, reported to significantly increase duodenal villi height and base width with no effect on crypt depth in broiler chicken [32]. In general, polyphenol-rich propolis extracts were found to strengthen the intestinal barrier in rats by activating AMP-activated protein kinase and extracellular signal-regulated kinases (ERK) signaling [72]. Xue et al. [73] reported that propolis supplementation to diabetic-induced rats modulated gut microbiota and improved intestinal epithelium tight and gap junctions.

The presented results are consistent and clearly show the negative effects of *E. coli* challenge on laying hen immunity. Cell mediated and humeral immunological parameters were significantly impaired with APEC challenge (Table 3). The negative effect of APEC on immune response was documented [74]. Mehaisen et al. [59] reported that the endotoxin shock induced by *E. coli* infection was responsible for the over-expression of apoptosis-related genes and increasing DNA damage of laying hen liver and brain cells. T-cells immuno-suppression, hypo-responsiveness and apoptosis were found to induce high ROS production in tumor cells [75]. The present results elicited severe inflammatory response to *E. coli* infection, with an increase in H/L ratio and high release of pro-inflammatory cytokines (TNF-α and IL-1β) (Table 2 and Table 3), that were reported to induce excessive production of ROS and exacerbate oxidative stress which negatively affects T-cell signaling, activation and proliferation [76,77,78]. Lymphoproliferative disorders of B cells were also reported to be mediate by high levels of ROS [79]. In the meantime, Wagner et al. [80] found correlation between humeral immune responses reduction and high frequencies of late-differentiated effector, memory effector and regulatory T-lymphocytes. Propolis supplementation significantly alleviates, to some extent, the negative effects of *E. coli* on laying immunity (Table 3). Furthermore, propolis supplementation to non-challenged hens significantly induced T- and B-lymphocytes proliferation. These effects may be mediated by the rich polyphenol content of propolis that helped in relieving ROS accumulation and inflammation to the extent that they, subsequently, activate T-cell proliferation [50]. These advantages of propolis on immune response can be justified by the capability of propolis and its bioactive compound to reduce inflammation and subsequently enhance immune response [35,65,81].

Leucocytes cell viability was significantly reduced by 29% in EC and by 12% in PR + EC groups (Figure 3a). These results can be justified by the endotoxin stress imposed by APEC which reported to induce DNA damage in the liver and brain cells and apoptosis in laying hens [59]. Besides, Foxo3 protein, a transcription factor that has been related to reduction of cell proliferation [82] and cell death [83], was over-expressed with the *E. coli* challenge (Figure 3b). It has been found that Foxo1/3 is expressed in different immune cell types with high expression in T- and B-lymphocytes and very low levels in macrophage [84]. They concluded that a cascade involving kinase Mst-Foxo/nuclear factor 2 (Nrf2) signaling pathway is important for attenuating ROS-induced immune cell damage and pathological states including infection diseases. Up regurlation of Foxo3 was reported to modulate helper T cell quiescence [85] and increase T-lymphocyts apoptosis [86]. Bi et al. [87] indicated that Foxo1, Foxo3 and Foxo4 are key genes in inhibiting lymphocyte cell proliferation of chicken infected with reticuloendotheliosis virus. It can be inferred that the over-expression of Foxo3 protein has a major contribution in the observed CV% reduction [88]. Taking altogether, these results suggested that propolis supplementation, attributed to its antioxidant properties, successfully managed to return the oxidation balance to immune cells by lowering Foxo3 expression and increasing CV%.

## 5. Conclusions

Pathogenic bacteria challenge is a major worldwide concern, especially with the increasing hazard of spreading antibiotic-resistance genes. The present results established solid evidence of the negative effects of *E. coli* administration on oxidative status, immune response and production performance of laying hens. Furthermore, APEC infection not only induced pro-inflammation response but also caused imbalance redox status and excessive ROS production. However, propolis supplementation at 0.1% of the diet improved oxidative status and immune response, simultaneously restoring the absent balance caused by *E. coli* infection. The positive influences of antioxidant compounds in the PR can reduce the MDA, IL-1β and corticosterone levels. Furthermore, PR was able to normalize the high expression level of Foxo3 and productive performance in layers exposed to EC. In addition, PR reduced inflammation reaction and enhanced leucocytes cells proliferation and viability. Thus, dietary PR can be used effectively as a natural product to bring back redox status balance and protect individuals from endotoxins, oxidative stress and immunosuppression imposed by pathogenic bacterial infection while avoiding the generating hazard of antibiotic resistant bacteria.

## Figures and Tables

**Table 1:** The effects of dietary propolis supplementation and *E. coli* challenge on layer productive performance.

Parameters	BD ^1^		BD + PR ^2^		SEM	*p*-Value		
	C ^3^	EC ^4^	PR + EC ^5^	PR ^6^		PR	EC	PR × EC
Egg Number/hen/7days	6.36 ^a^	4.08 ^c^	4.90 ^b^	6.76 ^a^	0.13	0.004	<0.0001	0.267
Egg weight, g	61.30 ^a^	53.60 ^c^	57.40 ^b^	63.20 ^a^	0.59	0.003	<0.0001	0.264
Feed intake, g/d	117.60 ^a^	88.60 ^c^	104.40 ^b^	115.80 ^a^	2.11	0.005	<0.0001	0.008
Feed conversion, kg/kg	2.11 ^b^	2.86 ^a^	2.63 ^a^	1.90 ^b^	0.08	0.073	<0.0001	0.953

Means in the same row with different superscripts differ significantly (*p* < 0.05). The presented productive parameters were measured as a mean of seven days post *E. coli* challenge. ^1^ laying hens received a basal diet; ^2^ laying hens received a basal diet supplemented with propolis at 1 g/kg feed; ^3^ laying hens received a basal diet and injected with saline (control); ^4^ laying hens received a basal diet and injected with *E. coli* (10^7^ colonies/hen); ^5^ laying hens received a basal diet supplemented with propolis at 1 g/kg feed and injected with *E. coli* (10^7^ colonies/hen) and ^6^ laying hens received a basal diet supplemented with propolis at 1 g/kg feed and injected with saline. Feed conversion was calculated as kg feed/kg egg mass.

**Table 2:** The effects of dietary propolis supplementation and *E. coli* challenge on levels of inflammatory biomarkers and antioxidant status in peripheral blood mononuclear cells (PBMCs), and plasma corticosterone concentration in laying hens (*n* = 10).

Parameters	BD ^1^		BD + PR ^2^		SEM	*p*-Value		
	C ^3^	EC ^4^	PR + EC ^5^	PR ^6^		PR	EC	PR × EC
TNF-α, pg/mL	94.68 ^c^	178.00 ^a^	155.50 ^b^	93.66 ^c^	4.79	0.026	<0.0001	0.039
IL-1β, ng/mL	0.29 ^c^	0.83 ^a^	0.52 ^b^	0.26 ^c^	0.03	<0.0001	<0.0001	0.0002
Cort, pg/mL	5.44 ^c^	14.86 ^a^	9.26 ^b^	3.28 ^c^	0.79	0.0002	<0.0001	0.044
MDA, μM/mL	2.00 ^b^	4.35 ^a^	2.36 ^b^	1.60 ^b^	0.43	0.014	0.002	0.086
TAC, μM/mL	3.97 ^b^	2.63 ^c^	3.31 ^bc^	7.29 ^a^	0.42	0.0002	<0.0001	0.006
SOD, μM/mL	355.6 ^b^	355.0 ^b^	386.0 ^ab^	425.0 ^a^	18.0	0.013	0.288	0.302

Means in the same row with different superscripts differ significantly (*p* < 0.05). ^1^ laying hens received a basal diet; ^2^ laying hens received a basal diet supplemented with propolis at 1 g/kg feed; ^3^ laying hens received a basal diet and injected with saline (control); ^4^ laying hens received a basal diet and injected with *E. coli* (10^7^ colonies/hen); ^5^ laying hens received a basal diet supplemented with propolis at 1 g/kg feed and injected with *E. coli* (10^7^ colonies/hen) and ^6^ laying hens received a basal diet supplemented with propolis at 1 g/kg feed and injected with saline. Abbreviations: PBMCs, peripheral blood mononuclear cells; SEM, standard error of mean; TNF-α, tumor necrosis factor α; IL-1β, interleukin 1β; Cort, corticosterone; MDA, malondialdehyde; TAC, total antioxidant capacity and SOD, super oxide dismutase.

**Table 3:** The effects of *E. coli* challenge and dietary propolis supplementation on immune performance in laying hens (*n* = 10).

Parameters	BD ^1^		BD + PR ^2^		SEM	*p*-Value		
	C ^3^	EC ^4^	PR + EC ^5^	PR ^6^		PR	EC	PR × EC
TWBC, ×10^3^/mL	56.86 ^b^	36.70 ^d^	47.70 ^c^	64.20 ^a^	2.34	0.001	<0.0001	0.445
H/L ratio	0.38 ^c^	0.98 ^a^	0.67 ^b^	0.32 ^c^	0.04	0.0007	<0.0001	0.009
SI T-lymphocytes	2.78 ^b^	1.02 ^d^	1.96 ^c^	3.64 ^a^	0.20	0.0004	<0.0001	0.845
SI B-lymphocytes	1.90 ^b^	0.94 ^c^	1.60 ^b^	2.80 ^a^	0.17	0.0003	<0.0001	0.487
Wattle thickness, mm	0.45 ^b^	0.36 ^c^	0.45 ^b^	0.54 ^a^	0.02	0.0006	0.0005	0.964

Means in the same row with different superscripts differ significantly (*p* < 0.05). ^1^ laying hens received a basal diet; ^2^ laying hens received a basal diet supplemented with propolis at 1 g/kg feed; ^3^ laying hens received a basal diet and injected with saline (control); ^4^ laying hens received a basal diet and injected with *E. coli* (10^7^ colonies/hen); ^5^ laying hens received a basal diet supplemented with propolis at 1 g/kg feed and injected with *E. coli* (10^7^ colonies/hen) and ^6^ laying hens received a basal diet supplemented with propolis at 1 g/kg feed and injected with saline. Abbreviations: TWBC, total white blood cells; H/L, heterophils to lymphocytes ratio and SI, stimulating index.

**Table 4:** The effects of dietary propolis supplementation and *E. coli* challenge on histomorphological measurements of small intestines in laying hens (*n* = 5).

Parameters	BD ^1^		BD + PR ^2^		SEM	*p*-Value		
	C ^3^	EC ^4^	PR + EC ^5^	PR ^6^		PR	EC	PR × EC
Villi height, μm	1960 ^c^	1905 ^c^	2083 ^b^	2272 ^a^	34.9	<0.0001	0.003	0.073
Crypt depth, μm	385 ^bc^	366 ^c^	406 ^b^	444 ^a^	10.8	0.0003	0.018	0.384
Villi/Crypts ratio	5.12	5.22	5.13	5.14	0.18	0.851	0.800	0.766

Means in the same row with different superscripts differ significantly (*p* < 0.05). ^1^ laying hens received a basal diet; ^2^ laying hens received a basal diet supplemented with propolis at 1 g/kg feed; ^3^ laying hens received a basal diet and injected with saline (control); ^4^ laying hens received a basal diet and injected with *E. coli* (10^7^ colonies/hen); ^5^ laying hens received a basal diet supplemented with propolis at 1 g/kg feed and injected with *E. coli* (10^7^ colonies/hen) and ^6^ laying hens received a basal diet supplemented with propolis at 1 g/kg feed and injected with saline.
